# Assessment of macular function in patients with non-vascularized pigment epithelial detachment

**DOI:** 10.1038/s41598-021-96151-8

**Published:** 2021-08-16

**Authors:** Marie Kitano, Asahi Fujita, Ryo Asaoka, Tatsuya Inoue, Tatsuaki Amari, Kayoko Komatsu, Motoshi Yamamoto, Asako Ogawa, Nobuyori Aoki, Masahiro Yamanari, Satoshi Sugiyama, Makoto Aihara, Satoshi Kato, Keiko Azuma, Maiko Maruyama-Inoue, Kazuaki Kadonosono, Ryo Obata

**Affiliations:** 1 Department of Ophthalmology, The University of Tokyo, Graduate School of Medicine, Tokyo, Japan; 2 Department of Ophthalmology, Seirei Hamamatsu General Hospital, Shizuoka, Japan; 3 Seirei Christopher University, Shizuoka, Japan; 4 Department of Ophthalmology and Micro-Technology, Yokohama City University, 4-57 Urafune, Minami-ku, Yokohama, Kanagawa 232-0024 Japan; 5 Tomey Corporation, Nagoya, Japan

**Keywords:** Diseases, Medical research

## Abstract

Non-vascularized pigment epithelial detachments (PED) are usually associated with dry age-related macular degeneration (AMD). In this study, we aimed to investigate the correlation between visual function and morphologic parameters. Seventeen eyes of eleven patients with non-vascularized AMD were enrolled. In addition to conventional optical coherence tomography (OCT), polarization-sensitive optical coherence tomography (PS-OCT) measurements were performed by evaluating the regularity of retinal pigment epithelium (RPE) entropy within the PED area. Retinal sensitivity was measured with MP-3 microperimetry, and retinal sensitivities within (RSin) and outside (RSout) the PED area were calculated. The relationship between OCT parameters and visual function was analyzed. As a result, there was a significant difference between the RSin and RSout (p < 0.001, Wilcoxon signed rank test). Moreover, RSin was significantly related to logMAR VA (p = 0.033, linear mixed model). The regularity of RPE entropy was significantly related to visual acuity and RSin (p = 0.00038, p = 0.031, linear mixed model), although neither the height nor area of PED correlated with visual function. Our results suggest that retinal sensitivity is significantly deteriorated within the PED area and RPE entropy measured with PS-OCT was closely related to visual function in eyes with non-vascularized PED.

## Introduction

Optical coherence tomography (OCT) was introduced approximately 30 years ago^1^. Since this introduction, there have been extraordinary advancements in various aspects of OCT, such as imaging speed, sensitivity, functional extensions, and available fields types. Nonetheless, a limitation still exists for the well-established intensity-based OCT in that tissue-specific contrast cannot be measured, and hence tissues cannot be directly differentiated. Polarization-sensitive optical coherence tomography (PS-OCT) is a relatively new technology that has been developed to generate tissue specific contrast by analyzing the polarization of light, in addition to the intensity. Previous studies have suggested immense possibilities exist for this new technique in various diseases, such as keratoconus^2–4^, glaucoma^5–9^, and macular diseases^10–19^. More specifically to our study, de Boer et al. recently reported the usefulness of PS-OCT in diagnosing age-related macular degeneration (AMD)^20^, because polarization scrambling is useful in identifying the concentration of melanin in the retinal pigment epithelium (RPE)^21,22^. Many additional reports have suggested the usefulness PS-OCT in the diagnosis of AMD lies in its ability to identify atrophic lesions in the RPE^13^, fibrotic scars^16,17,19^, and intraretinal migration of RPE^23^. However, these previous findings were limited in the structural assessment of AMD, and no report has yet been made to investigate the correlation between the assessment of RPE change by PS-OCT and visual function in patients with AMD.

In patients with AMD, pigment epithelial detachments (PED) are often observed as a precursor of the advancement of disease. This often occurs prior to the development of other retinal structural changes such as geographic atrophy^24^. Ogino et al. investigated the effect of the area and height of PED on retinal sensitivity in eyes with drusenoid PED using MP-1 microperimetry, and reported that these morphological changes were significantly correlated with retinal sensitivity^25^. In the current study, the association between the polarization of RPE evaluated by PS-OCT and visual function was investigated in eyes with non-vascularized PED; those with vascularized PED were excluded in order to directly evaluate the effect of changes in the polarization of RPE on retinal sensitivity, minimizing the effect of polarization changes in other retinal tissue. Moreover, in neovascular AMD, retinal sensitivity deteriorates not only due to PED, but also due to other factors such as choroidal neovascularization (CNV), macular edema, and subretinal fluid^26^.

In previous studies, the assessment of visual function in patients with AMD has been predominantly conducted through visual acuity (VA) evaluation which mainly reflects the function at the fovea. Recent reports have suggested the benefit of measuring visual function in a wider macular area using microperimetry, such as MP-3 microperimetry (Nidek, Japan)^27,28^. In the current study, visual function assessment was carried out using VA and the MP-3 microperimetry.

## Results

Seventeen eyes of eleven patients (7 men and 4 women) were retrospectively reviewed in the current study. Table 1 shows the baseline characteristics of these patients. Seven eyes had drusenoid PED and ten eyes had serous PED. The mean age (± standard deviation) was 69.7 ± 7.1 years. The values of the PED height (PEDh) and the PED area (PEDa) were 347.2 ± 171.3 um and 10.2 ± 7.5 mm^2^, respectively. The signal of the regularity of RPE entropy (RPEe) was evaluated as “continuous” in 8 eyes and “discontinuous” in 9 eyes.

**Table 1:** Baseline characteristics in the current study.

Variable	Mean ± SD [range]
Age (years)	69.7 ± 7.1 [58–80]
LogMAR VA	0.11 ± 0.20 [− 0.079 to 0.70]
CMT (μm)	195.1 ± 55.0 [109–281]
PEDh (μm)	347.2 ± 171.3 [108–709]
PEDa (mm^2^)	10.2 ± 7.5 [2.43–34.10]
Retinal sensitivity total (dB)	19.0 ± 6.0 [5.12–25.92]
RSin (dB)	15.7 ± 6.6 [3.93–24.67]
RSout (dB)	23.2 ± 4.6 [6.9–27.14]

*SD* standard deviation, *logMAR VA* logarithm of the minimum angle of resolution visual acuity, *CMT* central macular thickness, *PEDh* height of pigment epithelial detachment, *PEDa* area of pigment epithelial detachment, *RSin* retinal sensitivity within the pigment epithelial detachment, *RSout* retinal sensitivity outside the pigment epithelial detachment.

**Figure 1:**
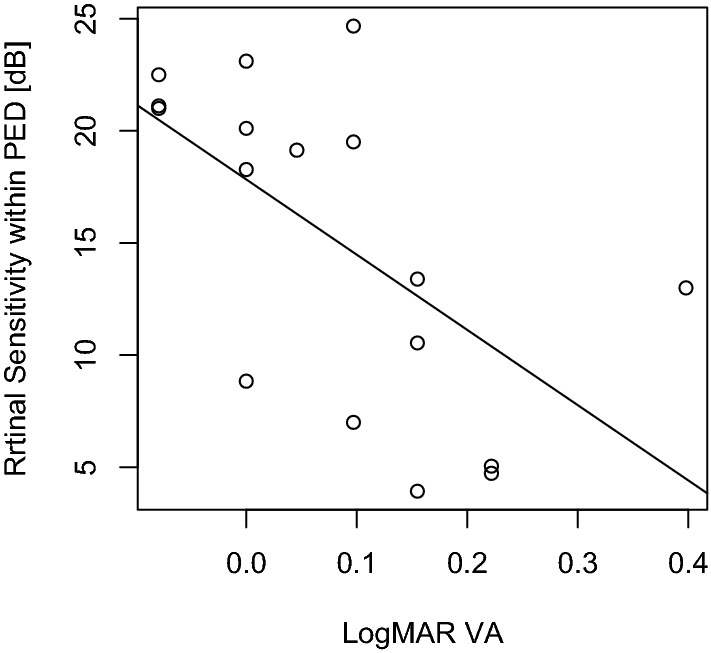
The correlation between logMAR VA and retinal sensitivity within the PED (RSin). *logMAR VA* logarithm of the minimum angle of resolution visual acuity, *PED* pigment epithelial detachment, *RSin* retinal sensitivity within the pigment epithelial detachment.

The retinal sensitivity was 19.0 ± 6.0 dB as an average of the whole 12°. Retinal sensitivity inside the PED area (RSin) was 15.7 ± 6.6 dB, and retinal sensitivity outside the PED area (RSout) was 23.2 ± 4.6 dB, respectively. There was a significant difference between RSin and RSout (p < 0.001, Wilcoxon signed rank test). RSin was significantly related to logMAR VA (Fig. 1, p = 0.033, linear mixed model). Univariate analysis between logMAR VA and the values of age, central macular thickness (CMT), PEDa, PEDh, and RPEe suggested that the RPEe was significantly correlated with logMAR VA (p = 0.00038, linear mixed model). There was no significant correlation between logMAR VA and the remaining variables (p > 0.05). Using the Akaike information criterion (AICc) model selection, only the RPEe was selected in the optimal model, indicating that a continuous RPEe signal was associated with better VA (Table 2, linear mixed model, AICc = − 26.1).

**Table 2:** Correlation between OCT parameters including RPE entropy and logMAR VA.

Variables	Univariate analysis			Optimal model^a^		
	Estimate	SE	p value	Estimate	SE	p value
Age	0.0053	0.0051	0.32	NS	NS	NS
CMT	0.00071	0.00054	0.21	NS	NS	NS
PEDa	− 0.0044	0.0046	0.36	NS	NS	NS
PEDh	0.00016	0.00017	0.37	NS	NS	NS
RPEe	0.19	0.042	**0.00038**	0.19	0.042	**0.00038**

*OCT* optical coherence tomography, *RPE* retinal pigment epithelium, *logMAR VA* logarithm of the minimum angle of resolution visual acuity, *SE* standard error, *NS* not selected, *CMT* central macular thickness, *PEDh* height of pigment epithelial detachment, *PEDa* area of pigment epithelial detachment, *RPEe* the entropy of retinal pigment epithelium.

^a^Multivariate analysis with model selection.

**Figure 2:**
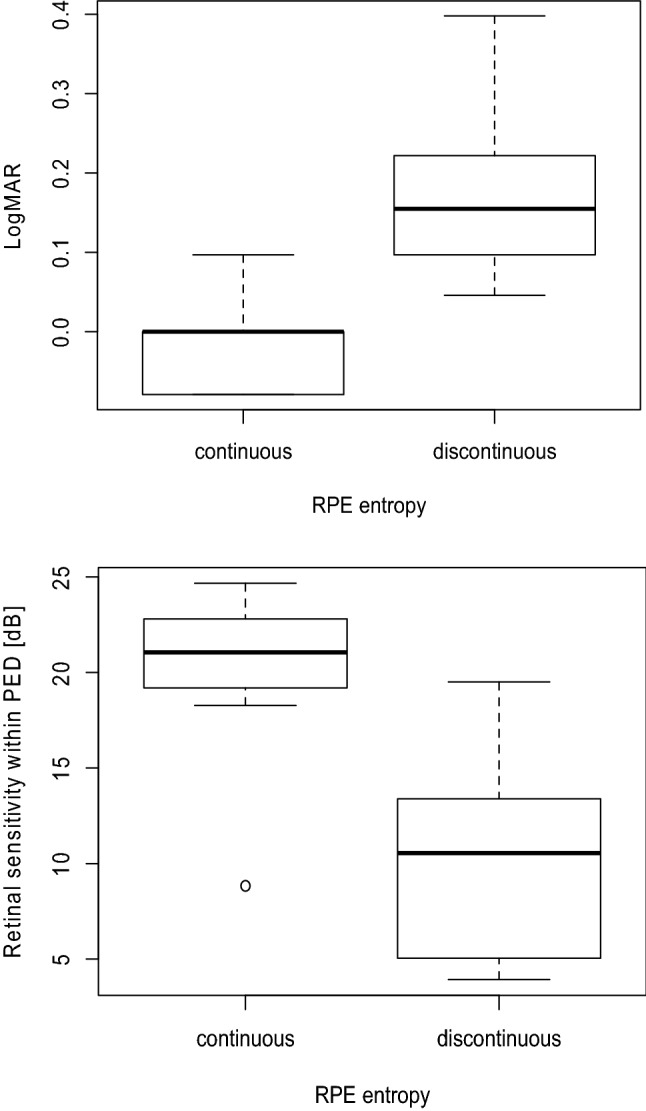
Box plots comparing the visual function of eyes with continuous and discontinuous RPE entropy. The visual function of eyes with continuous RPE entropy in the PED (continuous) is compared to the visual function of eyes with discontinuous RPE entropy (discontinuous). There were significant differences in logMAR VA (**A**) and retinal sensitivity (**B**) between two groups. *RPE* retinal pigment epithelium, *PED* pigment epithelial detachment, *logMAR VA* logarithm of the minimum angle of resolution visual acuity.

Univariate analysis between RSin and the values of age, CMT, PEDa, PEDh, and RPEe suggested that only RPEe was significantly correlated with RSin (Table 3, p = 0.031, linear mixed model). Among age, CMT, PEDa, PEDh, and RPEe, only RPEe was selected as the optimal model for RSin, as a result of the AICc model selection. (Table 3, linear mixed model, AICc = 105.5). There were significant differences in logMAR VA and RSin between continuous and discontinuous RPEe within the PED region (Fig. 2A, p = 0.00038; Fig. 2B, p = 0.031; linear mixed model).

**Table 3:** Correlation between OCT parameters and retinal sensitivity within PED.

Variables	Univariate analysis			Optimal model^a^		
	Estimate	SE	p value	Estimate	SE	p value
Age	− 0.21	0.33	0.54	NS	NS	NS
CMT	− 0.034	0.017	0.084	NS	NS	NS
PEDa	0.30	0.13	0.056	NS	NS	NS
PEDh	− 0.0069	0.0060	0.28	NS	NS	NS
RPEe	− 3.63	1.27	**0.031**	− 3.63	1.27	**0.031**

*OCT* optical coherence tomography, *PED* pigment epithelial detachments, *NS* not selected, *CMT* central macular thickness, *PEDh* height of pigment epithelial detachment, *PEDa* area of pigment epithelial detachment, *RPEe* the entropy of retinal pigment epithelium.

^a^Multivariate analysis with model selection.

In subgroup analysis, there were no significant differences in age, logMAR VA and RSin between drusenoid and serous PED (p = 0.99, p = 0.99, p = 0.89, respectively, Wilcoxon rank sum test). Furthermore, there was a significant difference in PEDh between two groups (p = 0.033) but no significant difference was observed in PEDa (p = 0.15). In serous PED subgroup, PEDh was significantly associated with both logMAR VA and RSin (p = 0.030, p = 0.0088, linear mixed model). On the other hand, there was no significant correlation between PEDh and visual function in drusenoid PED.

## Discussion

In the current study, we investigated the relationship between morphologic parameters and visual function in patients with non-vascularized PED. As a result, retinal sensitivity inside the PED was significantly deteriorated compared with that outside the PED. Moreover, we found that RPE entropy measured with PS-OCT was closely correlated with both retinal sensitivity measured with MP-3 and logMAR VA.

Our present results suggested that the height and the area of non-vascularized PED were not related to retinal sensitivity and visual acuity; these findings are inconsistent with a previous report^25^. In the previous study, macular function in 18 eyes with drusenoid PED was analyzed using MP-1 microperimetry. As a result, the height and area of the drusenoid PED were significantly associated with retinal sensitivity within the central 4 and 8°. One possible reason for this discrepancy is the difference in patients’ background because our present study included eyes with serous PED in addition to drusenoid PED. However, our current result suggested that neither PEDh nor PEDa was associated with visual functions in drusenoid PED subgroup. It might be presumably due to the small sample size in this study and further research is needed to clarify the correlation between OCT parameters and visual function in eyes with non-vascularized PEDs.

Compared to the conventional OCT findings, our result suggested that PS-OCT was useful to predict visual functions in eyes with non-vascularized PED. This may be because PS-OCT measurement sharply reflects the RPE function by detecting RPE melanocytes, whereas conventional OCT can measure only morphological abnormalities such as RPE aperture or intraretinal RPE migration and not any functional deterioration. Furthermore, it is advantageous to use depolarized images even when simply clarifying morphological evaluation of RPE. Miura et al. previously reported that PS-OCT is superior to conventional OCT in detecting focal RPE defect in the serous PED with AMD^23^, supporting this idea.

**Figure 3:**
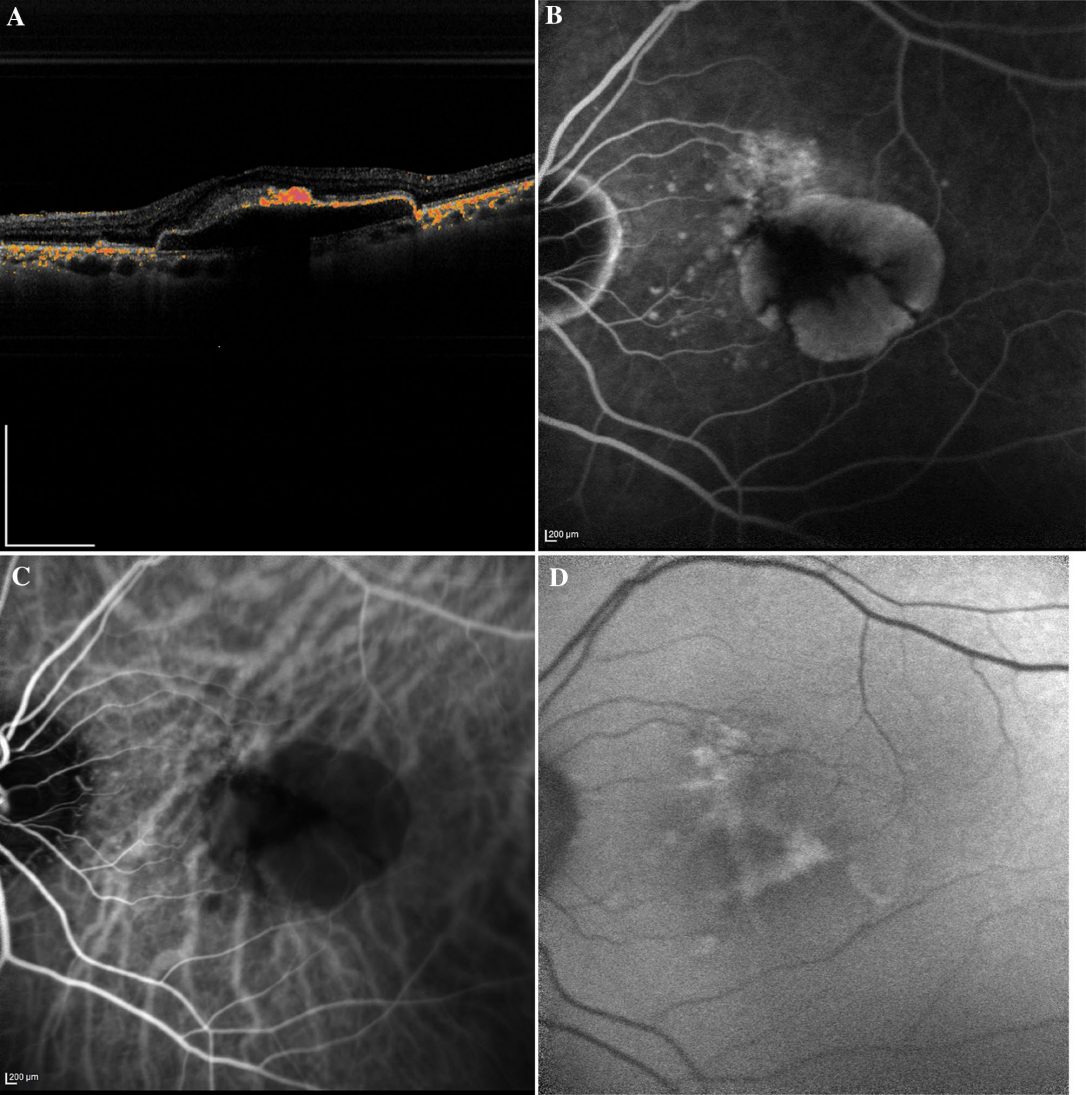
Depolarized image and FA, ICGA and FAF findings in nonvascularized PED. (**A**) The depolarized image of the B-scan showed the discontinuity of RPE entropy. Of note, no obvious signs of RPE atrophy were observed at the PED in FA (**B**), ICGA (**C**) and FAF (**D**). *FA* fluorescein angiography, *ICGA* indocyanine green angiography, *FAF* fundus autofluorescence, *PED* pigment epithelial detachment.

Drusenoid PEDs sometimes develop geographic atrophy, which is the main cause of progressive visual loss in dry AMD. In the Age-Related Eye Disease Study (AREDS) report, 19% of eyes with drusenoid PED progressed to geographic atrophy by 5 years follow-up^29^. It is possible that the decreased depolarization (discontinuous RPE entropy) might sharply detect RPE atrophy at the PED. We recently reported that there was a decrease in depolarization at the PED around the RPE aperture, which was mainly found in drusenoid PED eyes^30^. Retinal sensitivity was significantly decreased in the area surrounding the RPE aperture and was associated with the degree of depolarization, demonstrating the presence of RPE atrophy in the decreased depolarization area. PS-OCT provides tissue-specific 3-dimensional information, therefore it might be superior to other ophthalmic examinations in estimating RPE atrophy in eyes with nonvascular PED. Actually, some patients with nonvascularized PED demonstrated no obvious sign of RPE atrophy in fluorescein angiography (FA), indocyanine green angiography (ICGA) and fundus autofluorescence (FAF) but the discontinuous RPE entropy in PS-OCT (Fig. 3).

The present study has some limitations including its retrospective nature and small sample size. In addition, this study was cross-sectional, and, therefore, the change in PED over time cannot be assessed. Our present results suggested that visual function was more closely related to the RPE change evaluated by PS-OCT in eyes with AMD and non-vascularized PED. However, we could not determine whether the change in RPE entropy in patients with PED precedes other ophthalmic findings, such as fundus autofluorescence. Finally, quantitative analysis of RPE entropy was not performed in the current study. It would be of interest to examine whether the quantification of RPE entropy enables to predict visual function in the future.

In conclusion, the decreased visual function in AMD eyes with non-vascularized PED was significantly correlated with the discontinuous RPE entropy. These findings indicate the usefulness of PS-OCT in evaluating macular function associated with structural changes in non-vascularized PED.

## Methods

The Research Ethics Committee of the Graduate School of Medicine and Faculty of Medicine at The University of Tokyo approved this single-center, cross-sectional study. The study protocol adhered to the tenets of the Declaration of Helsinki. Written informed consent was obtained from each patient.

We reviewed the clinical records of patients who were diagnosed with non-vascularized AMD with associated PED at the University of Tokyo Hospital. The following information was apparent from this evaluation:

1. Drusenoid PEDs are usually found in dry AMD and diagnosed due to the presence of confluent drusen; no obvious CNV is noted on FA, ICGA or OCT angiography.
2. Serous PEDs are observed as sharply demarcated elevations of the RPE; no CNVs were observed in patients with serous PED.

All patients underwent comprehensive ophthalmic examinations including visual acuity and OCT assessment. Spectral domain OCT (Spectralis, Heidelberg Engineering) was used to measure the PEDa, PEDh, and CMT. In eyes with multiple PEDs, the PEDh was measured in the largest PED and the PEDa was calculated as the sum of all PED areas. CNV was not detected by FA, ICGA, FAF and OCTA measurements in all studied eyes. Moreover, no signs of other retinal diseases, such as CSC, were observed during follow-up period.

**Figure 4:**
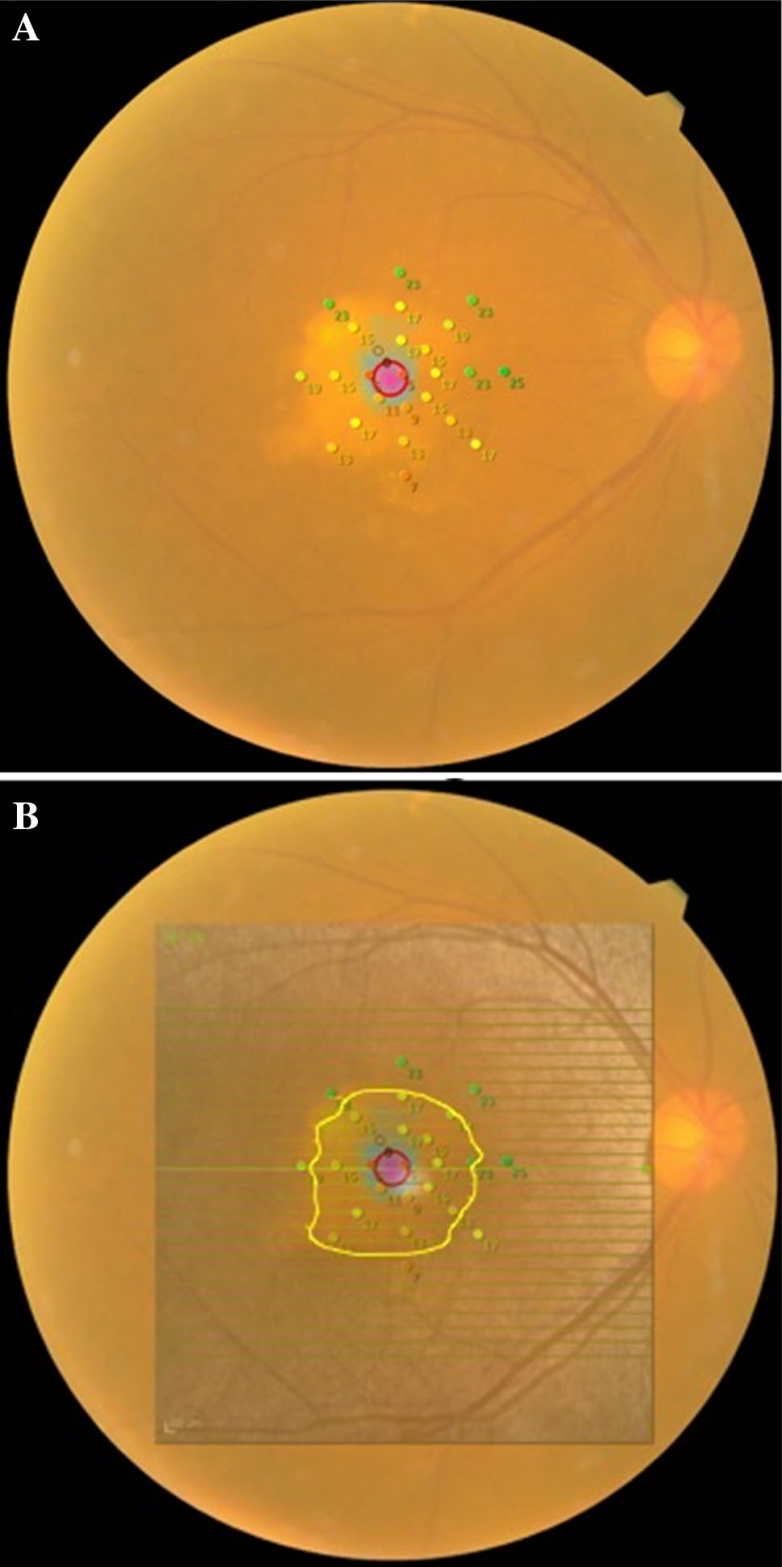
MP-3 microperimetry of the eye. (**A**) Representative image of MP-3 microperimetry in an eye with PED. (**B**) Superimposing OCT image on the retinal sensitivity measured with MP-3 microperimetry. Yellow line indicates the border of PED. PED, pigment epithelial detachment.

Retinal sensitivity was measured using fundus-monitored microperimetry (MP-3, Nidek, Japan). A 4-2-staircase strategy with Goldmann III-sized stimuli was used with 25 stimulus locations within 12°, as previously described (Fig. 4A)^31^. To investigate the correlation between visual function and OCT parameters, retinal sensitivity was superimposed on OCT images for all subjects (Fig. 4B). Retinal sensitivities inside (RSin) and outside (RSout) the PED area were calculated.

**Figure 5:**
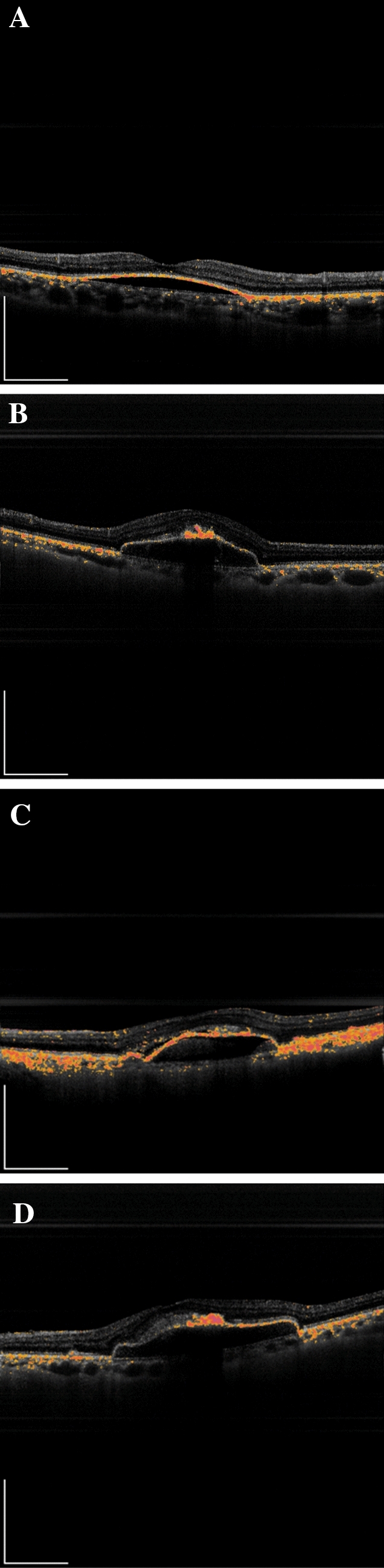
Representative PS-OCT images in eyes with PED. Representative PS-OCT B-scan images in eyes with PEDs. (**A**) serous PED with continuous RPE entropy (**B**) serous PED with discontinuous RPE entropy (**C**) drusenoid PED with continuous RPE entropy (**D**) drusenoid PED with discontinuous RPE entropy. *PS-OCT* polarization-sensitive optical coherence tomography, *PED* pigment epithelial detachment, *RPE* retinal pigment epithelium.

PS-OCT measurements were performed using a clinical prototype for retinal imaging (Tomey Corp, Nagoya, Japan). The system of PS-OCT is based on the technology of swept source OCT and the light source was a wavelength-swept laser with a center wavelength of 1050 nm. The optical interferometer was also used to detect the elements of Jones matrix, which mathematically characterizes the polarization property of the target. The area of 6 × 6 mm was scanned using 512 A-lines × 512 B scans in 3.3 s. By averaging the intensities of the Jones-matrix elements, polarization independent intensity image was obtained from each eye. Polarization scrambling or depolarization was parameterized as entropy. To investigate the regularity of RPE entropy (RPEe), the depolarization images measured with PS-OCT were assessed in all examined eyes (Fig. 5). Using the horizontal scan PS-OCT images, we investigated whether the depolarization signal was continuous along with RPE throughout the PED area for each eye. Two examiners (MK and AF) evaluated the RPEe in eyes with non-vascularized PED. The RPEe was assessed as “continuous” or “discontinuous”. In cases where the second examiner did not agree with the first examiner, a panel discussion (MK, AF and TI) was held to draw a conclusion.

The correlation between visual functions (visual acuity [VA] and RSin) and the OCT parameters (age, PEDh, PEDa, CMT, and RPEe) was analyzed using univariate and multivariate linear regression. In addition, using AICc model selection, we investigated which parameter was the best explanatory variable for visual functions. In multivariate regression models, the degrees of freedom decreases with an increasing number of variables; hence, it is recommended to use model selection methods to improve the model fit by removing redundant variables rather than by performing simple multivariate regression analysis, particularly when the number of explanatory variables is large^32,33^. The AIC is an established statistical measure used to evaluate the relationship between variables, and the AICc denotes the corrected AIC, providing an accurate estimation even when the sample size is small^34^. Thus, the optimal model for logMAR VA or RSin was obtained from 2^5^ patterns with five variables (age, PEDh, PEDa, CMT, and RPEe). All statistical analyses were performed using the statistical programming language R (R version 3.1.3; The Foundation for Statistical Computing, Vienna, Austria).

### Summary statement

This study aimed to investigate the correlation between visual function and morphologic parameters, including polarization-sensitive optical coherence tomography. Our results suggest that retinal sensitivity significantly deteriorated within the pigment epithelial detachment area. Furthermore, retinal pigment epithelium entropy was closely related to visual function in eyes with non-vascularized pigment epithelial detachments.
